# Supplementary material for: Oncogenic human papillomavirus and anal microbiota in men who have sex with men and are living with HIV in Northern Taiwan
Source: PLoS One. 2024 Dec 31;19(12):e0304045. doi: 10.1371/journal.pone.0304045 (PMC11687658; doi:10.1371/journal.pone.0304045)
Supplement: S1 Table — (DOCX) [file pone.0304045.s001.docx]

Supplemental Table S1: Results of the permutational multivariate analysis of variance (adonis function) and the multivariate homogeneity of group dispersions analysis (betadisper function)

| **Comparisons** | **Beta diversity indices** | **Adonis P** | **Betadisper P** |
| --- | --- | --- | --- |
| Gr1 vs Gr2 | Unweighted UniFrac | 0.3556 | 0.1798 |
|  | Weighted UniFrac | 0.6923 | 0.6753 |
|  | Variance adjusted weighted UniFrac | 0.7343 | 0.6194 |
|  | GUniFrac with alpha 0.5 | 0.5415 | 0.966 |
|  | NMDS on Bray-Curtis distance | 0.06394 | 0.4595 |
| Gr1 vs Gr3 | Unweighted UniFrac | 0.0989 . | 0.7822 |
|  | Weighted UniFrac | 0.4466 | 0.988 |
|  | Variance adjusted weighted UniFrac | 0.1299 | 0.08192 |
|  | GUniFrac with alpha 0.5 | 0.2957 | 0.8851 |
|  | NMDS on Bray-Curtis distance | 0.01499 * | 0.6633 |
| Gr1 vs Gr4 | Unweighted UniFrac | 0.8601 | 0.2617 |
|  | Weighted UniFrac | 0.5485 | 0.9401 |
|  | Variance adjusted weighted UniFrac | 0.1598 | 0.3277 |
|  | GUniFrac with alpha 0.5 | 0.5045 | 0.7363 |
|  | NMDS on Bray-Curtis distance | 0.7622 | 0.4346 |
| Gr1 vs Gr5 | Unweighted UniFrac | 0.2248 | 0.03896 * |
|  | Weighted UniFrac | 0.6693 | 0.6334 |
|  | Variance adjusted weighted UniFrac | 0.3626 | 0.7153 |
|  | GUniFrac with alpha 0.5 | 0.4116 | 0.2657 |
|  | NMDS on Bray-Curtis distance | 0.08492 | 0.08791 |
| Gr1 vs Gr6 | Unweighted UniFrac | 0.01499 * | 0.1009 |
|  | Weighted UniFrac | 0.07193 | 0.8352 |
|  | Variance adjusted weighted UniFrac | 0.01798 * | 0.06494 |
|  | GUniFrac with alpha 0.5 | 0.03097 * | 0.7093 |
|  | NMDS on Bray-Curtis distance | 0.000999 *** | 0.7752 |
| 6 groups | Unweighted UniFrac | 0.1169 | 0.01399 * |
|  | Weighted UniFrac | 0.3057 | 0.8601 |
|  | Variance adjusted weighted UniFrac | 0.1918 | 0.5724 |
|  | GUniFrac with alpha 0.5 | 0.08691 | 0.6024 |
|  | NMDS on Bray-Curtis distance | 0.001998 ** | 0.1628 |
