# Supplementary material for: Oncogenic human papillomavirus and anal microbiota in men who have sex with men and are living with HIV in Northern Taiwan
Source: PLoS One. 2024 Dec 31;19(12):e0304045. doi: 10.1371/journal.pone.0304045 (PMC11687658; doi:10.1371/journal.pone.0304045)
Supplement: S1 File — (PDF) [file pone.0304045.s002.pdf]

HPV Questionnaire

1	Year/month of birth	□□YY□□MM
2	Education	□1)primary。□2)junior high。□3)high school。□4)vocational school。□5)college。□6)post graduate。
3	Marriage	□1)never。□2)married。□3)divorced。□4)separated。□5)widowed。
4	Year/month of HIV	□□YY□□MM
5	Current smoker	□1)No。□2)Yes(□□pack/day、lasting□□y)
6	Current betelnut chewer	□1)No。□2)Yes(□□nut/day、lasting□□y)
7	Current alcohol consumer?	□1)No。□2)Yes(□occasional、□often、□everyday)
8	Numbers of lifetime sexual partner	□□□□persons
9	Numbers of sexual partners in 1/2 years	□□□□persons
10	Gender of your sexual partners	□1)men、□2)women、□3)both。
11	Practice receptive anal sex	□1)every time、□2)often、□3)occasional、□4)rare、□5)never。
12	Condom use during anal sex	□1)every time、□2)often、□3)occasional、□4) rare 、□5)never。
13	Practice oral sex	□1)every time、□2)often、□3)occasional、□4) rare 、□5)never。
14	Condom use during oral sex	□1)every time、□2)often、□3)occasional、□4) rare 、□5)never。
15	Met sexual partners on the web	□1)every time、□2)often、□3)occasional、□4) rare 、□5)never。
16	Sex for exchange of money	□1)every time、□2)often、□3)occasional、□4) rare 、□5)never。
17	STI in 1/2 years	□No。□Yes。
18	Circumcision	□No。□Yes。
19	Substance use in 1/2 years	□1)every time、□2)often、□3)occasional、□4) rare 、□5)never。
20	Injection drug use in 1/2 year	□1)every time、□2)often、□3)occasional、□4) rare 、□5)never。
21	Chemsex	□No。□Yes。

22	HAART use	□No。□Yes。
23	Latest Lab (YY/MM)	CD4________________, HIV viral load_________________
24	HPV vaccination	□No。□Yes。
