# Supplementary material for: Oncogenic human papillomavirus and anal microbiota in men who have sex with men and are living with HIV in Northern Taiwan
Source: PLoS One. 2024 Dec 31;19(12):e0304045. doi: 10.1371/journal.pone.0304045 (PMC11687658; doi:10.1371/journal.pone.0304045)
Supplement: Attachment — Submitted filename: gabri.DOCX [file pone.0304045.s003.DOCX]

Plos one user name ALatini-532

The manuscript by Chu et al describes the results of a study that performed 16S analysis in anal samples collected from MSM-LWH, characterized in terms of cytology and HPV infection. This is a quite underexplored field of research. The study seems to be well designed and well conducted, although histology is missing and only cytology results are available. The following issues should be addressed:

- Line 110: specify how HPV test results are provided (HPV16/18 together and all the other types as a pool)
- The protocol for DNA extraction ad purification from anal samples is completely missing; this should be added since it is a crucial step in high-throughput microbial sequencing
- Lines 174-194: avoid this detailed description of the study groups; instead, provide a brief description together with a table or a flow-chart to report HPV and cytology results as well as the number of subjects for each group
- Lines 199-201: the p values reported in the text are different from those shown in Figure 1
- Lines 217-223: move this part to Materials and Methods section
- Lines 223-232: I would suggest summarizing in a Supplementary Table the results of the comparisons between the different groups in terms of beta diversity; the text is difficult to follow and the significant information hard to capture
- Lines 281-304: I would suggest to reduce this part and to move it to the Introduction, since it is not strictly related to the discussion of the study results
- Line 351: I would avoid the use of the word “AIN” since the authors did not provide results for histology
- Lines 352-355: this is not a study limit; it is a different finding compared to others; thus, this part should be moved above
- Lines 357-359: rephrase this sentence
- Line 360: what did the authors mean with “they should be followed”? How? The study did not explore or show any correlation between microbiome composition and risk of pre-cancer in these patients
- Replace “ASCUS or higher grade” with ASCUS+ throughout the text
- Replace “PLWH who are MSM” with MSM-LWH throughout the text
- Avoid repeating the details of the study subjects every time the corresponding group is mentioned; once the groups have been described, they can be mentioned using the group number

Table 1

Provide the number (%) of participants who were on cART, and how many of these had undetectable VL
