# Supplementary material for: Oncogenic human papillomavirus and anal microbiota in men who have sex with men and are living with HIV in Northern Taiwan
Source: PLoS One. 2024 Dec 31;19(12):e0304045. doi: 10.1371/journal.pone.0304045 (PMC11687658; doi:10.1371/journal.pone.0304045)
Supplement: Attachment — Submitted filename: Response_to_Reviewers.docx [file pone.0304045.s004.docx]

| 1. Queries | Answers |
| --- | --- |
| 2. The manuscript by Chu et al describes the results of a study that performed 16S analysis in anal samples collected from MSM-LWH, characterized in terms of cytology and HPV infection. This is a quite underexplored field of research. The study seems to be well designed and well conducted, although histology is missing and only cytology results are available. | Thank you for your helpful comments. In this study, we strongly recommended high-resolution anoscopy to obtain histology results for patients with HPV 16/18 or cytology yielding ASCUS+. Less than 20% of the patients actually visited proctologists. The patients’ reluctance to undergo anoscopy motivated us to explore non-invasive biomarkers. |
| 3. Line 110: specify how HPV test results are provided (HPV16/18 together and all the other types as a pool) | Line 125: The results for HPV16/18 are provided independently and together, along with pooled results for types 31, 33, etc. |
| 4. The protocol for DNA extraction ad purification from anal samples is completely missing; this should be added since it is a crucial step in high-throughput microbial sequencing | 16S rRNA was purified from anal samples using the QIAamp Fast DNA Stool Mini Kit (QIAGEN, Germany). This detail has been included under the heading “Targeted amplicon library preparation and sequencing”. |
| 5. Lines 174-194: avoid this detailed description of the study groups; instead, provide a brief description together with a table or a flow-chart to report HPV and cytology results as well as the number of subjects for each group | Thank you for your feedback. A flow chart (Fig. 1) has been created accordingly, which is referenced in lines 220. |
| 6. Lines 199-201: the p values reported in the text are different from those shown in Figure 1 | Line 226-227: We apologize for any confusion. The p-values are as follows:  0.033  0.022  0.053 |
| 7. Lines 217-223: move this part to Materials and Methods section | Thank you for your suggestion. Lines 156-170: Alpha and beta diversity information was moved to the Methods section. |
| 8. Lines 223-232: I would suggest summarizing in a Supplementary Table the results of the comparisons between the different groups in terms of beta diversity; the text is difficult to follow and the significant information hard to capture | Thank you for your helpful suggestion. Supplementary Table S1 has been created accordingly. |
| 9. Lines 281-304: I would suggest to reduce this part and to move it to the Introduction, since it is not strictly related to the discussion of the study results | Thank you for the suggestion. We have revised the text as follows: Lines 73-87  Ravel et al. [21] described bacterial vaginosis-associated bacteria (community state type IV) as a group characterized by the depletion of Lactobacillus spp. and the presence of anaerobic species like Gardnerella, Megasphaera, Sneathia, and Prevotella. Lactobacilli provide broad-spectrum protection in the vagina [22]. An imbalance, such as a depletion of Lactobacillus and an increase in other microbiota, can lead to histological alterations in the vaginal mucosa and cervical epithelium [23,24]. A previous meta-analysis showed that bacterial vaginosis is associated with higher rates of HPV infection (odds ratio 1.43, 95% CI 1.11–1.84) [25], suggesting that a Lactobacillus-depleted microbiome may contribute to HPV persistence in the cervix. In addition, bacterial vaginosis may involve reactive oxygen species that cause DNA damage, facilitating HPV integration and malignant transformation [23]. Women living with HIV (PLWH) were found to have cervical lesions with increased bacterial diversity and reduced Lactobacillus spp. [26,27], similar to women without HIV [13,19]. However, studies on anal lesions, HPVs, and anal mucosa microbiota are limited [20, 28,29]. |
| 10. Line 351: I would avoid the use of the word “AIN” since the authors did not provide results for histology | Line 348: "AIN" was replaced with "precancerous anal cytology" per your suggestion. Thank you. |
| 11. Lines 352-355: this is not a study limit; it is a different finding compared to others; thus, this part should be moved above | Thank you for pointing this out. We moved this section discussing the contribution of Fusobacterium and Sneathia to HPV-related lesions from the Limitations to the Discussion: lines 339-342. |
| 12. Lines 357-359: rephrase this sentence | We have rephrased the sentence accordingly. Please refer to lines 358-361. |
| 13. Line 360: what did the authors mean with “they should be followed”? How? The study did not explore or show any correlation between microbiome composition and risk of pre-cancer in these patients | Lines 356-361: The study showed PLWH with CD4+ T cell counts of <500 cells/µL, anal ASCUS+, and detectable HPV16/18 have low bacterial diversity and a relative abundance of *Enterobacteriaceae, Ruminococcus*, and *Bacilli* compared to other participant groups. We concluded that understanding the interaction among immunocompromised hosts, oncogenic HPVs, and microbiota is essential, and the contribution of these factors to anal precancerous lesions needs more in-depth exploration. |
| 14. Replace “ASCUS or higher grade” with ASCUS+ throughout the text | Thank you for your comment. “ASCUS or higher grade” was replaced with "ASCUS+" throughout the text. |
| 15. Replace “PLWH who are MSM” with MSM-LWH throughout the text | “PLWH who are MSM” was replaced with “MSM-LWH” throughout the text. |
| 16. Avoid repeating the details of the study subjects every time the corresponding group is mentioned; once the groups have been described, they can be mentioned using the group number | We replaced the details with the group numbers accordingly. |
| 17. Table 1 18. Provide the number (%) of participants who were on cART, and how many of these had undetectable VL | We added the details to Table 1 per your suggestion. |
| 19. In this study “Oncogenic human papillomavirus and anal microbiota in men who have sex with men and are living with HIV in Northern Taiwan”, HPV DNA tests and citology results in anal samples collected Jun 2018 and Dec 2021 in 291 persons living with HIV (PLWH), mainly MSM were analyzed and 16S sequencing was performed to characterize the anal microbiome. Data on the interaction of HPV, HIV and resident commensal bacteria are needed and may be useful in determining the relative risk of lesion progression to anal cancer precursors. Despite the merits of the study, a clearer presentation of the results, additional information and more in-depth discussion are needed in a revised version. | Thank you for your valuable comments. We have revised the presentation of the results for clarity and provided more details to the discussion. |
| 20. Method: A paragraph on the Bioinformatic analysis methods should be added to explain the tests that are listed in Results: e.g. UniFrac, PcoA on GuniFrac, Non-metric Multidimensional Scaling (NMDs), Bray-Curtis distance (VST), Adonis tests and the Betadisper test. | The details were added under the subheading: “*16s rRNA gene sequence analysis*” at lines156-172 in the *Methods* section:  “Alpha diversity was measured, which indicated the richness and evenness of bacterial taxa within a community, calculated by using the estimate_richness function from the phyloseq package with observed, chao index, Shannon, and Simpson methods; Beta diversity, which reflects the extent of variation in microbial community composition between the two groups, using Principal Co-ordinates Analysis (PcoA) on unweighted Unique Fraction (UniFrac) and weighted UniFrac, PcoA on variance-adjusted weighted UniFrac; and PcoA on Generalized Unique Fraction (GUniFrac) with alpha 0.5. UniFrac distances were calculated using the GUniFrac package (v1.1) to assess the community dissimilarity between groups [40]. Non-metric Multidimensional Scaling (NMDs) on Bray-Curtis distance (VST), Adonis tests (to examine whether composition between groups was similar), and the Betadisper test (to test the homogeneity of dispersion between groups, which is an assumption of Adonis) was calculated using DESeq2 package (V1.26.0) [33]. Microbiota enrichment analysis was conducted using the Linear Discriminant Analysis (LDA) effect size (LefSe) method with the Wilcoxon–Mann–Whitney test (at α = 0.05) and logarithmic LDA score of more than 2 [41] and visualized as a cladogram using GraPhlAn [42]. |
| 21. The paragraph HPV genotyping should be renamed to HPV detection and it should be more clearly stated that individual results for HPV 16 and HPV 18 are given, along with a simultaneous, pooled result for the other oncogenic HPVs. Was high-resolution anoscopy (HRA) performed after an ASCUS+ result? | Thank you for your insightful comments and the question. We have renamed the paragraph to "HPV detection" and clarified the reporting of individual results for HPV 16 and HPV 18, as well as the pooled results for other oncogenic HPVs. Additionally, we have included information on the performance of high-resolution anoscopy (HRA) following an ASCUS+ result. In this study, we strongly suggested HRA to obtain the histology results for those who had HPV 16/18 or cytology-yielding ASCUS+. However, less than 20% of the patients actually visited proctologists. The willingness to undergo invasive procedures is a significant barrier. The patients’ reluctance to undergo anoscopy motivated us to explore non-invasive biomarkers. |
| 22. Results The presentation of results should be improved. For example, the distribution of samples in the six groups could be presented in a table. | We have added a flow chart to explain the distribution of samples across the 6 groups. |
| 23. The individual results for HPV 16, HPV 18 and other oncogenic HPVs should be reported in a table or text and whether positivity was statistically associated with ASCUS+ results. Moreover, how many patients were diagnosed with anal SIL should be reported | We appreciate your suggestion. At lines 201-204, we have added the individual results for HPV 16, HPV 18, and other oncogenic HPVs to the Results section, along with statistical associations with ASCUS+ results. Furthermore, we have reported the number of patients diagnosed with anal SIL in Table 2.  **Text Revision:**  **Results:** Among the anal samples from 291 participants, 36 (12.4%) yielded HPV 16, 38 (13.1%) yielded HPV 18, and 190 (65.3%) yielded pooled results for 12 other oncogenic HPVs. |
| 24. Discussion: Lines 281-282: “The interaction between microbiota, HPV acquisition and persistence, and the development of cervical cancer in women has been researched in detail.” I would suggest to modify this sentence in: the vaginal microbiota has been studied in detail, but the interactions with HPV acquisition and persistence and cervical cancer risk are still not fully understood. Accordingly, I would suggest to shorten the description of the vaginal microbiota community state types (CSTs) that are well known (lines 283-295) and to focus on studies (if any) about the anal mucosa microbiota in women. | Lines 73-87: We have revised the sentence to clarify that while the vaginal microbiota has been studied in detail, the interactions of HPV acquisition and persistence with cervical cancer risk are still not fully understood. Additionally, we have shortened the description of the vaginal microbiota CSTs and included information about studies on the anal mucosa microbiota in women. Following a suggestion from a previous reviewer, we have moved this paragraph to the introduction section for better context. |
| 25. venes 306-308: “In the pathophysiology of infectious diseases, pathogenic colonization and infection involve dynamic interactions between microbes and the microbiome, host, and environmental factors [39]. This biology is also applicable to PLWH who are MSM” The sentences “This biology is also applicable to PLWH who are MSM” should be rephrased, e.g. in This biology also applies to the anal mucosal environment of PLWH who are MSM. | Lines 209-299: We appreciate your suggestion. We have rephrased the sentence to better convey the intended meaning: This biology could be also applied to the anal mucosal environment of MSM-LWH [43]. |
| 26. At line 322: “homogeneity of the dispersion also differed between the groups...” This should be rephrased for a better comprehension of this concept. | Thank you for your suggestion. We have rephrased the sentence to improve clarity and comprehension.  **Text Revision:**  **Lines 308-310:** "The variability within each group also differed, indicating differences in the spread or dispersion of microbial communities between the groups." |
| 27. At lines 323-325: “Enterobacteriaceae, Proteus, Morganellaceae, Mycoplasma, Sutterella, Ruminococcus, and Bacilli were significantly abundant in PLWH whose CD4+ T cell counts were < 500 cells/µL and who had anal cytology yielding ASCUS or higher grades with detectable HPV 16/18 (group 1).” As this sentence implies a comparison, other group(s) should be mentioned. | Line 311: We added “than groups 2,3,4, and 6” to clarify the comparison. |
| 28. At lines 328-331: “HIV infection also frequently correlates with increased tissue and circulating measures of inflammation, such as CD14, IL-6, and CD38+HLA-DR+CD8+ T cells in the gut [41], as well as increases in traditionally pathogenic bacteria such as Enterobacteriaceae, including Morganellaceae and Proteus [42,43].” The sentence should be reworded to more clearly relate the results of the references mentioned here [41-43] to the results of this study. | Lines 313-324: We rephrased the section as follows: “Dinh et al. studied 21 PLWH and found the relative abundance of intestinal Proteobacteria, Gammaproteobacteria, Enterobacteriales, and Enterobacteriaceae was significantly enriched compared to HIV-negative controls [45]. Dubourg et al. studied 31 PLWH and showed that PLWH has an increased relative abundance of gut Gammaproteobacteria compared to HIV-negative cases [46]. Previous researchers have conducted comprehensive reviews highlighting increased inflammatory biomarkers, including CD14, IL-6, and CD38+HLA-DR+CD8+ T cells in the gut, as well as increases in traditionally pathogenic bacteria such as Enterobacteriaceae, including Morganellaceae and Proteus [46,47]. Similar to the current study, we also demonstrated a higher relative abundance of Enterobacteriales and Enterobacteriaceae in group 1 compared to other groups. However, these previous studies did not examine the role of HPVs or the differences in anal cytology findings.” |
| 29. The same applies to the sentences at lines 342-343: “Our findings are consistent with those of Ron et al., who described Ruminococcus-predicting HSIL [40]. In contrast, a decreased abundance of Ruminococcus was previously noted in the guts of PLWH [46].” | Lines 333-339: We rephrased the section as follows: “Ron et al. studied 128 MSM, of whom 47 (36.7%) had biopsy-proven HSIL, and found that Ruminococcus is a predictor of HSIL [44]. Although the richness of this anaerobic commensal, Ruminococcus was found to decrease in the gut samples of PLWH [50], caution should be exercised when it is found in abundance at anal sites, particularly in the Ruminococcaceae NK4A214 group [44]. The above-mentioned anal bacterial ecology changes with the presence of oncogenic HPVs and the transformation of the anal epithelium.” |
| 30. and to the sentences at lines 353-354 (limitations): “Third, previous studies have mentioned the contribution of Fusobacterium and Sneathia to HPV-related lesions [22,39], which could not be proven in this study” Ref 39 (Pathogens, microbiome and the host: emergence of the ecological Koch's postulates) is a review; hence, the specific study reporting about Fusobacterium and/or Sneathia should be specified and discussed. | We have revised the sentences and moved to lines 340-342 (discussion) for better clarity and specificity. Reference 39 has been changed to the original article by 43. (Lee et al. Association of the vaginal microbiota with human papillomavirus infection in a Korean twin cohort. PLOS ONE. 2013;8: e63514. doi: 10.1371/journal.pone.0063514.) |
| 31. If applicable, we recommend that you deposit your laboratory protocols in [protocols.io](http://protocols.io/) to enhance the reproducibility of your results. Protocols.io assigns your protocol its own identifier (DOI) so that it can be cited independently in the future. For instructions see: <https://journals.plos.org/plosone/s/submission-guidelines#loc-laboratory-protocols>. Additionally, PLOS ONE offers an option for publishing peer-reviewed Lab Protocol articles, which describe protocols hosted on [protocols.io](http://protocols.io/). Read more information on sharing protocols at <https://plos.org/protocols?utm_medium=editorial-email&utm_source=authorletters&utm_campaign=protocols>. 32. Critically, additional clarity in the methods section is necessary and the data should be presented more concisely. Some of the statistical methodology might also be suboptimal. | The 16S rRNA analysis pipeline using publicly available information.  <https://github.com/ycl6/16S-rDNA-V3-V4>  We have rewritten the sub-sections of the Methods, subheading “targeted amplicon library preparation” and “16s rRNA gene sequence analysis”, to improve the clarity. In addition, we rechecked the property of statistical methods. Lastly, we made the results section more concise. |
| 33. . Please ensure that your manuscript meets PLOS ONE's style requirements, including those for file naming. The PLOS ONE style templates can be found at <https://journals.plos.org/plosone/s/file?id=wjVg/PLOSOne_formatting_sample_main_body.pdf> and <https://journals.plos.org/plosone/s/file?id=ba62/PLOSOne_formatting_sample_title_authors_affiliations.pdf> | We have ensured that the formatting guidelines are met. |
| 34. 2. We noticed you have some minor occurrence of overlapping text with the following previous publication(s), which needs to be addressed: <https://academic.oup.com/jid/article/224/7/1247/6129308?login=false> 35. In your revision ensure you cite all your sources (including your own works), and quote or rephrase any duplicated text outside the methods section. Further consideration is dependent on these concerns being addressed. | Thank you for pointing out the overlap with the previous publication (<https://academic.oup.com/jid/article/224/7/1247/6129308?login=false>). We have carefully reviewed the overlapping sections and made necessary revisions to address this issue. We have rephrased the duplicated text and ensured proper citation of all sources, including our own works.  iThenticate has been used for overlapping checks before resubmission. |
| 36. Page 4 Line 79 listing inclusion and exclusion criteria for invitation to participate explicitly might be helpful. Currently the Line 79 implies MSM was an inclusion criteria but 9 participants failed this (P8L166)? | Line 192: The 9 participants who are heterosexual misunderstood the inclusion criteria, and they were therefore excluded from the final analysis. |
| 37. P6 L123– 124 how many indexes were used in one run? If all 291 samples were assessed by 16rRNA seq this means that batches were made. Was batch effect investigated and how was it addressed? | Lines 140-143: We have a maximum of 96 indexes per run, but the actual number will depend on the samples of the run. The batch effect is assessment and correction using the DESeq2 package in R. |
| 38. P6 L127 which flow cell and reagents (cartridge) were used (v2/v3)? | Details were added to lines 140-142: “Finally, the libraries were normalized and pooled in an equimolar ratio by Nextera XT DNA Library Preparation Kit (Illumina) and sequenced using an Illumina Miseq instrument MiSeq Reagent Kit V3 (600 cycle) (Illumina Inc., San Diego, CA, USA).” |
| 39. P7 L129 are the raw sequences deposited in a sequence repository? | Thanks for your reminder. The ASV was uploaded as a supplementary file. |
| 40. P7 L138 was some preprocessing / filtering done on the pyloseq object or during preceeding steps? Ie normalization or filtering of taxa? | Line 148: Filtering, dereplication, and learning the error rates were performed using dada2 before creating the pyloseq object. The detailed pipeline can be found at <https://github.com/ycl6/16S-rDNA-V3-V4>. |
| 41. P7 L140-144 last two paragraphs lack version and or references for the packages used. | The version information and references were added under the subheading “16S rRNA gene sequence analysis” in the Methods section: Line 140-143: the libraries were normalized and pooled in an equimolar ratio by Nextera XT DNA Library Preparation Kit (Illumina equimolar ratio by Nextera XT DNA Library Preparation Kit (Illumina) and sequenced using an Illumina Miseq instrument MiSeq Reagent Kit V3 (600 cycle) (Illumina). |
| 42. P7 L150 testing every combination with t-test is inappropriate without multiple testing correction. | Thank you for pointing this out. We used the Wilcoxon–Mann–Whitney test when applicable (Line 170).” |
| 43. Why was Deseq not used since phyloseq is supposed to be analysed with deseq for differential abundance <http://joey711.github.io/phyloseq-extensions/DESeq2.html> | Line 168: DESeq2 was used for the analysis of the difference between groups in beta-diversity |
| 44. P7 L151 6 groups for analysis seems excessive? Possibly this level of detail is appropriate for the supplement tables. It is difficult to confirm but the Figure 1 groups appear to be not mutually exclusive (Groups 3 and 6 appear to have the most participants and those 2 groups do not specify the CD4 cell counts unlike all other groups where the cutoff of 5000 cells was employed.) | In this study, we have tried several approaches including 2 groups (divided by cytology [Normal and ASCUS compared to SIL]), 2 groups (HPV16/18 compared to noHPV16/18), 3 groups (SIL compared to ASCUS, Normal/inflammation), and 6 groups (oncogenic HPVs + level of cytology), and the current 6 groups (HPV16/18, level cytology and CD4+T cells counts <500 cells/uL) for comparisons. These results are available as supplementary material. We intended to explore the signature microbiota for participants with CD4+T cell counts <500 cells/uL+HPV16/18+ASUCS+ and found these 22 MSM-LWH did have uniqueness. |
| 45. P8 L165 it might be more informative if failed cytology and failed HPV exclusions were shown separately | We added this information to the flow chart (Fig 1) and lines 191-193: “10 (2.9%) failed cytology interpretation, 28 (8.3%) failed HPV genotype tests, and 9 (2.7%) were heterosexual PLWH.” |
| 46. P8 Table 1 “educational year” is worded sub optimally | We revised the phase accordingly. |
| 47. P8 Table 1 it might be necessary to specify what smoking and drinking represent? Were some guidelines placed in the questionnaire that might be important for interpreting what “yes” answers mean? | The data on smoking and drinking represent the current status of the participants. In the questionnaire, participants were asked to report their daily cigarette consumption and the duration of their smoking history. Regarding drinking, participants selected one of three options: seldom, often, or every day. The Mandarin version of the questionnaire, which includes these details, is provided in the supplementary material.  We believe these clarifications will help in accurately interpreting the "yes" answers related to smoking and drinking behaviors. Thank you for your attention to this detail. |
| 48. P9 Table 1Was the normality of numerical variables tested? Can all reliably be summarized with mean(SD)? | We added the median (25^th^,75^th^ quartile) if values were not normally distributed. |
| 49. P9 Table 1 were there missing answers in the questionnaires? | The electronic questionnaire is designed to not miss any item. |
| 50. P8-9 Table 1 contains excessive white space. | We revised Table 1 accordingly – thank you for pointing this out. |
| 51. P10 data regarding HPV/cytology and other laboratory measures should be provided in a table for the groups (plus total) discussed instead of text only. | We have detailed the description of HPV across various levels of anal cytology findings in Table 2. Thank you for your suggestion to improve the presentation of our results. |
| 52. P10 L195 the materials and methods do not adequately introduce the alpha and beta diversity metrics used in the results section. | Lines 156-170: The details were added under a subheading: “*16s rRNA gene sequence analysis*” in *methods,* line: Alpha diversity was measured, which indicated the richness and evenness of bacterial taxa within a community, calculated by using the estimate_richness function from the phyloseq package with observed, chao index, Shannon, and Simpson methods; Beta diversity, which reflects the extent of variation in microbial community composition between the two groups, using Principal Co-ordinates Analysis (PcoA) on unweighted Unique Fraction (UniFrac) and weighted UniFrac, PcoA on variance-adjusted weighted UniFrac; and PcoA on Generalized Unique Fraction (GUniFrac) with alpha 0.5. UniFrac distances were calculated using the GUniFrac package (v1.1) to assess the community dissimilarity between groups [40]. Non-metric Multidimensional Scaling (NMDs) on Bray-Curtis distance (VST), Adonis tests (to examine whether composition between groups was similar), and the Betadisper test (to test the homogeneity of dispersion between groups, which is an assumption of Adonis) was calculated using DESeq2 package (V1.26.0) [33]. Microbiota enrichment analysis was conducted using the Linear Discriminant Analysis (LDA) effect size (LefSe) method with the Wilcoxon–Mann–Whitney test (at α = 0.05) and logarithmic LDA score > 2 [41], and visualized as a cladogram using GraPhlAn [42]. |
| 53. Figure 1 shows some numbers but no context is provided what 500 or 1000 means | The numbers are the measure of alpha diversity in different methods. |
| 54. P11 L199-201 using t-test for the pairwise comparison of 6 groups is inappropriate. If the data is normally distributed then an ANOVA test with suitable post test should be done. | Line 170: We used the Wilcoxon test, considering it as a better alternative to the t-test when the normal distribution of the differences between paired individuals cannot be assumed. |
| 55. P11 L201/201 total number of participants in each group should be shown. | Line 220: Fig. 1 (flow chart) has been provided. The case numbers for each group are shown in the content and figures. |
| 56. Please submit your revised manuscript by Aug 23 2024 11:59PM. If you will need more time than this to complete your revisions, please reply to this message or contact the journal office at [plosone@plos.org](mailto:plosone@plos.org). When you're ready to submit your revision, log on to <https://www.editorialmanager.com/pone/> and select the 'Submissions Needing Revision' folder to locate your manuscript file. | Thank you again for the opportunity to review the manuscript, as well as for all his insightful suggestions. |
