# Supplementary material for: Oncogenic human papillomavirus and anal microbiota in men who have sex with men and are living with HIV in Northern Taiwan
Source: PLoS One. 2024 Dec 31;19(12):e0304045. doi: 10.1371/journal.pone.0304045 (PMC11687658; doi:10.1371/journal.pone.0304045)
Supplement: Attachment — Submitted filename: Response to reviewers.docx [file pone.0304045.s005.docx]

**Response to reviewers**

| Additional editor’s comments | Replies |
| --- | --- |
| Please review your reference list to ensure that it is complete and correct. If you have cited papers that have been retracted, please include the rationale for doing so in the manuscript text, or remove these references and replace them with relevant current references. Any changes to the reference list should be mentioned in the rebuttal letter that accompanies your revised manuscript. If you need to cite a retracted article, indicate the article’s retracted status in the References list and also include a citation and full reference for the retraction notice. | We have reviewed and checked the reference list. |
| P7 Line 143-144 grammatically incorrect and possibly misplaced. Batch effect would be compensated after trimming and denoising. | We have corrected the grammar and revised the sentence to “Batch effects were compensated after trimming and denoising” and moved it to an appropriate location on page 7, line 150. |
| Page 8 Line 173 statistical analysis section  The authors reply “Line 170: We used the Wilcoxon test, considering it as a better alternative to the ttest when the normal distribution of the differences between paired individuals cannot be assumed.“  However, there are several issues with this revision.  1)The revision deleted the mention of t-test from Statistical analysis section at line 177 leaving only chi-square test mentioned  2) The Figure 2 p values, the Figure 2 legend, or the text at P12 L227 remains unchanged suggesting that it still shows the old t-test individual p values. The test used to obtain the p values is undeclared and inconsistent with the paragraph Statistical analysis  3) The comparisons between the six groups presented at Figure 2 cannot be considered paired individuals and Wilcoxon test cannot be considered as appropriate for these comparisons | We apologize for the confusion regarding the statistical methods. To clarify:   1) The p-values in the figures were derived from Wilcoxon tests (the default method in the analysis package). Student t-tests were never used in this study. We have now explicitly mentioned the use of the Wilcoxon–Mann–Whitney test at both line 173 (in the "16s rRNA gene sequence analysis" section) and line 170 of the Statistical analysis section. 2) We have corrected Figure 2, its legend, and the text at P12 L227 to reflect the use of Wilcoxon tests. The statistical methods are now explicitly stated in the Figure 2 legend and at P13 L243. 3) In Figure 2, we have shown a comparison of group 1 (PLWH with CD4+ T cell counts < 500 cells/µL, ASCUS+, and detectable HPV 16/18) with each of the other groups individually using the Wilcoxon test, rather than comparing all six groups simultaneously. The Kruskal–Wallis test was not used. |
| P12 Table 2 footer would benefit from listing the 12 other HPV types as a footnote | We have added the 12 additional HPV types as a footnote in Table 2. |
| Supplementary table 1 was uploaded twice. (once as Table_S1.docx and once as S1_table.docx) on pages 40 and 41 of the combined PDF | Thank you for pointing this out. We have corrected the duplication of Supplementary Table 1. |
